# Pre- and Post-Race Intestinal Microbiota in Long-Distance Sled Dogs and Associations with Performance

**DOI:** 10.3390/ani10020204

**Published:** 2020-01-25

**Authors:** Kristoffer Relling Tysnes, Inga Leena Angell, Iselin Fjellanger, Sigrid Drageset Larsen, Silje Rebekka Søfteland, Lucy J. Robertson, Ellen Skancke, Knut Rudi

**Affiliations:** 1 Department of Paraclinical Sciences, Faculty of Veterinary Medicine, Norwegian University of Life Sciences, P.B. 369 Sentrum, 0102 Oslo, Norway; kristoffer.tysnes@nmbu.no (K.R.T.); iselinfjellanger@outlook.com (I.F.); Sigrid_dl91@hotmail.com (S.D.L.); silje.softeland@outlook.com (S.R.S.); lucy.robertson@nmbu.no (L.J.R.); 2 Department of Chemistry, Biotechnology and Food science (IKBM), Norwegian University of Life Sciences, 1430, Box 5003 Ås, Norway; inga.angell@nmbu.no; 3 Department of Companion Animal Clinical Sciences (SportFaMed), Faculty of Veterinary Medicine, Norwegian University of Life Sciences, 0102 Oslo, Norway; ellen.skancke@nmbu.no

**Keywords:** sled dog, microbiota, dysbiosis, 16S rRNA gene

## Abstract

**Simple Summary:**

The impact of the gut microbiota on endurance performance remains unresolved. Here, we present an association between endurance performance and gut microbiota dysbiosis in sled dogs. We present evidence that normobiosis-associated bacteria prevent the outgrowth of dysbiosis-associated bacteria during the race.

**Abstract:**

Although our understanding of the role of the gut microbiota in different diseases is improving, our knowledge regarding how the gut microbiota affects functioning in healthy individuals is still limited. Here, we hypothesize that the gut microbiota could be associated with sled dog endurance-race performance. We investigated the gut microbiota in 166 fecal samples from 96 Alaskan Huskies, representing 16 teams participating in the 2016 Femund Race (400 km) in Norway, relating the microbiota composition to performance and metadata derived from questionnaires. For 16S rRNA gene sequencing-derived compositional data, we found a strong negative association between *Enterobacteriaceae* (dysbiosis-associated) and *Clostridium hiranonis* (normobiosis-associated). The teams with the best performances showed both the lowest levels of dysbiosis-associated bacteria prior to the race and the lowest change (decrease) in these bacteria after the race. Taken together, our results support the hypothesis that normobiosis-associated bacteria are involved in resilience mechanisms, potentially preventing growth of *Enterobacteriaceae* during the race.

## 1. Introduction

The gut microbiota can be considered an organ, providing essential functions to the host, including short-chain fatty acid (SCFA) production, immune modulation, and protection against some pathogens. The role of the intestinal microbiota in health and disease has been of increasing interest, with associations being explored in both intestinal and extra-intestinal diseases [1]. In healthy humans and dogs, the gut microbiota is in a stable normobiotic state [2] and is resilient towards changes. However, following severe perturbations, such as extensive antibiotic treatment, the gut microbiota may not return to normobiosis but, rather, remain dysbiotic [3]. Dysbiosis is characterized by loss of the normal functions of the microbiota [2,4]. In particular, increased oxygen tension has been related to the dysbiotic state [5].

Dysbiosis in both dogs and humans has been associated with gut-related diseases, such as inflammatory bowel disease (IBD) [2,4,6]. However, how the normobiotic microbiota is associated with good health remains less clear. Good endurance performance could be used as a proxy for good health; indeed, potential associations between endurance performance and composition of the gut microbiota have been reported in humans [7,8,9] but not yet in dogs. Thus, long-distance racing in sled dogs might represent a good model for investigating associations between gut microbiota signatures and good health.

Endurance racing with dogs is a recreational sport of increasing popularity in North America and various European countries, including Norway. Several long-distance sled dog races are held annually, such as the 400 km Femund Race in Norway, and both professional and amateur teams participate. These races exert an enormous physical load on the dogs [10], and their ability to perform under this extreme pressure may be affected by their microbiomes; at the same time, the race itself may affect the composition of the dogs’ microbiomes. Although information about the effects of long-distance racing on microbiota composition in sled dogs is scarce, a study from Alaska reported a pronounced alteration in the fecal microbiome after a 300-mile race, compared with that observed in Labradors participating in field trials in a restricted location [11].

The aim of our study was to characterize the intestinal microbiota and level of dysbiosis in sled dogs participating in the 400 km Femund Race by means of 16S rRNA gene sequencing and fecal score measurements, with relation to performance and other factors, such as age, dietary information, and team. The dysbiosis-associated bacteria were measured using a newly developed dysbiosis index for dogs. The index is based on seven key bacterial taxa, with relation to both inflammation and bile acid metabolism [6].

We present results showing an overall major reduction of dysbiosis-associated bacteria in the participating dogs, with the team having the overall lowest degree of dysbiosis-associated bacteria showing the best performance. These results indicate the importance of gut dysbiosis in endurance performance.

## 2. Methods

### 2.1. Study Design

The Femund Race is arranged annually in Mid-Eastern Norway during February. Teams compete in either the 400 km (8 dogs per team) or the 600 km (12 dogs per team) trail. The 400 km race lasts about 2 days, with a total racing time of about 30 h and rest time of about 15 h. The race course for both trails starts and ends in the town of Røros. The race area is dominated by forest and mountain plateaus, with temperatures down to −40 °C (for more information, visit: http://www.thefemundrace.com/).

The study population consisted of dogs/teams participating in the 400 km Femund Race in 2016. Mushers (*n* = 81) listed in the official Femund Race participant list were randomized in Microsoft Excel, and the first 20 mushers in the randomized list were contacted during November–December 2015 and all agreed to participate in our study. Four teams withdrew from the study before the race, leaving 16 mushers that were willing to participate, of which 14 responded to the questionnaire. Each team consisted of 8 dogs, and the mushers were asked to choose six of them to be included in the study. The dogs were classified by breed; gender; and age (<1 year, 1–3 years, and >3 years), and further information regarding kennel size, housing, type of food, and amount of pre-race exercise was collected through a questionnaire (Table S1). Mushers were also asked to give details about feeding routines.

### 2.2. Sample Collection and Storage

Fecal samples for microbiota analyses were collected from up to six dogs per team the day before and immediately after finishing the race (not all dogs were available for sampling; see Results section). Collection was done by direct rectal swab using FecalSwab^TM^, a collection and transport system containing a Cary–Blair medium (Copan Diagnostics Inc., Murrieta, CA, USA). Samples were immediately frozen and stored at −20 °C and transported in a cool box with cooling elements within a maximum of 8 h, before being stored at −80 °C until further processing at the Norwegian University of Life Sciences (NMBU). The samples were not thawed before DNA extraction.

In addition, fecal samples (three from each included dog) were collected by their owner and sent to NMBU both two weeks before and two weeks after the race. These samples were examined for parasites using standard techniques: modified McMaster and immunomagnetic separation (IMS) immunofluorescence assay test (IFAT) for the *Giardia duodenalis* and *Cryptosporidium* spp. [12]. The fecal score was determined based on consistency using a modified version of the Purina Fecal Scoring System from 1 to 4 (1 = liquid stool, 2 = soft nonformed stool, 3 = soft but formed stool, and 4 = firm and formed stool).

Ethical Approval and Consent to Participate: This study was approved by the Ethical Committee for Animals at the Norwegian University of Life Sciences (reference number 15/04947) according to the International Association of Veterinary Editors Consensus “Author Guidelines on Animal Ethics and Welfare for Editors”. Consent for Publication: All participating mushers were informed about the study aims and methods and signed a “consent to participate” form, which informed them that we intended to make the results publicly available, before being included in the study.

### 2.3. Microbiome Analysis

DNA was extracted from the rectal swabs directly after thawing using a mag midi kit (LGC Genomics, UK) according to the manufacturer’s instructions in an automated KingFisher flex system (Thermo Fisher Scientific, Waltham, MA, USA). To determine taxonomic composition, library construction and sequencing of resulting amplicons of the V3–V4 region of the 16S rRNA were done, as previously described [13]. The resulting amplicon reads were processed (demultiplexing, merging, primer removal, quality filtering, dereplicating, operational taxonomic unit (OTU) clustering, and filtering of chimeras) using a standard procedure associated with USEARCH 8.0 software [14] in the QIIME environment [15], with Silva [16] as the reference database for taxonomic assignments. Read counts for all OTUs in each sample were arranged into an OTU table and then rarefied to 10,000 sequences to obtain a uniform sequencing depth.

The level of dysbiosis-associated bacteria was determined by utilizing a recently developed dysbiosis index for dogs based on 7 bacterial subgroups, including *Faecalibacterium, Turicibacter, Streptococcus, Escherichia coli, Blautia, Fusobacterium,* and *Clostridium hiranonis* [6]. The sequencing-based dysbiosis index was calculated based on summing the log10 number of reads for OTUs classified to genera/species positively associated with dysbiosis, with subsequent subtraction of the log10 number of reads belonging to OTUs classified to genera/species negatively associated with dysbiosis. *E. coli* was represented by *Enterobacteriaceae* due to lack of resolution in the 16S rRNA gene. The average dysbiosis index within teams was used to investigate correlations between dysbiosis and performance. 

Raw reads from the 16S rRNA gene sequencing are available in the National Center for Biotechnology Information (NCBI) sequence read archive (SRA) database with accession number SRP148740.

### 2.4. Statistics

Statistical analyses were performed using Minitab software (Minitab Inc., State College, PA, USA). Multivariate statistical analyses were done using PLS_Toolbox (Eigenvector Research, Inc.; Seattle, WA, USA) in the MATLAB programming environment (MathWorks, Inc.; Natick, MA, USA).

## 3. Results

### 3.1. Overall Composition of the Microbiota

**Figure 1:**
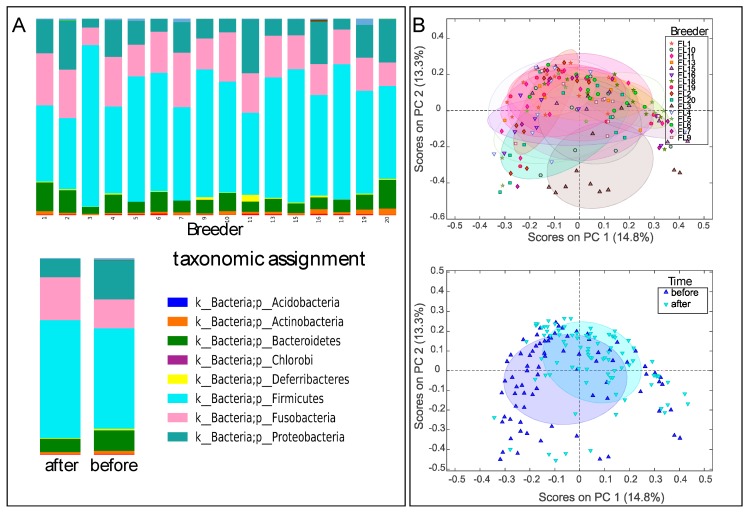
Phylum-level distribution (**A**) and Bray–Curtis diversity (**B**) of the microbiota. Upper panels for A represent team distributions, while lower panels represent after and before the race. The 50% confidence level is labelled for Bray–Curtis distribution. The beta-diversity plots were generated using principal coordinates analysis (PCoA) ordination.

In total, samples from 96 dogs representing 16 teams were obtained for fecal microbiota analysis. Some dogs were not available for sampling, either pre or post-race or both. Samples from 79 dogs before the race and 87 dogs after the race were included in the microbiota analysis. All samples yielded 10,000 sequences or more. There were relatively large individual differences in the fecal microbiota across the sled dogs, with a dominance of *Clostridium* and *Fusobacteria* species. There was also a relatively low correlation in microbiota composition before and after the race when comparing the data for the same dogs (Spearman rho of 0.62 ± 0.10 (mean ± std), *n* = 70 comparisons), which was only marginally higher than for the comparison of different dogs (0.54 ± 11 (mean ± std), *n* = 71 comparisons). Despite large individual divergences, team-specific differences were observed, in addition to differences before and after the race (Figure 1A). Beta diversity clustering was observed for teams, in addition to clustering associated with samples taken before and after the race (Figure 1B).

**Figure 2:**
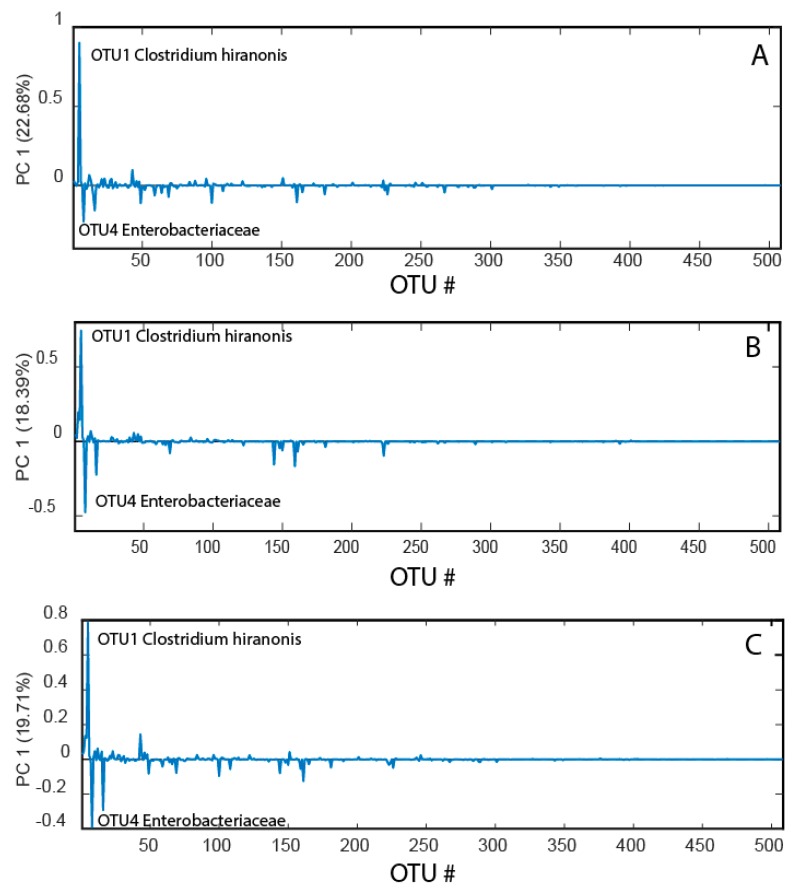
Bacterial loadings before the race (**A**), after the race (**B**), and changes from before to after the race (**C**). The bacterial loadings were derived from the first principal component in a PCoA model for the mean-centered operational taxonomic unit (OTU) table.

Data compression with the principal coordinates analysis (PCoA) showed similar loading patterns for before, after, and changes from before to after the race (Figure 2). In all cases, the main pattern was that *Clostridium hiranonis* showed a strong negative association with *Enterobacteriaceae.*

### 3.2. Diversity Measures

ANOVAs confirmed the clustering structure in the data related to the teams, in addition to before and after the race. The team effect was determined by 13 principal components (PCs), indicating team-specific signatures of the fecal microbiota composition (effect 14.0, *p* < 0.0001). The effect of the race (before and after) was determined by a single PC (effect 3.6, *p* < 0.0001), indicating similar effects across all teams.

There were no systematic differences in species richness across teams, but there was a significant (*p* = 0.001, *t*-test) decrease from 133.4 ± 32.2 to 113.2 ± 20 (mean ± std) in species richness from before to after the race. The race did not, however, affect alpha diversity measures Simpsons 1-D or Shannon H (results not shown).

### 3.3. Level of Dysbiosis-Associated Bacteria

**Figure 3:**
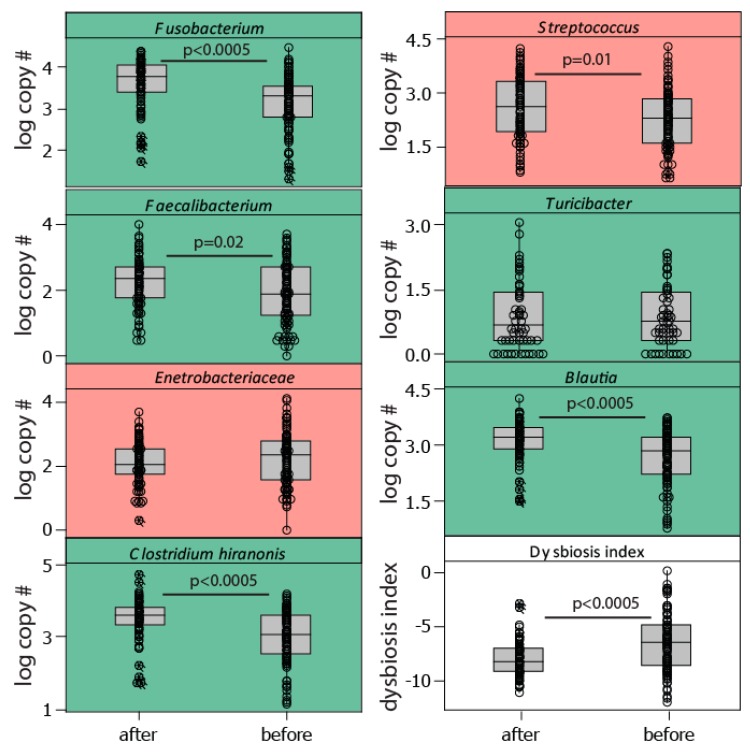
Bacterial taxa included in the dysbiosis index. The dysbiosis index was calculated as the sum of the log10 copy number for bacteria positively associated with the dysbiosis index (marked in red), while subtracting those negatively associated (marked in green). The association with dysbiosis has previously been determined [6]. Abbreviations: after—after the race, before—before the race.

We found a significant effect of the teams (H = 35.64, *p* = 0.002; Kruskal Wallis) on the level of the dysbiosis index and a decrease in the dysbiosis index from before the race to after the race (H = 17.3, *p* < 0.0005; Kruskal Wallis). This was manifested by a considerable post-race increase in the bacteria *Fusobacterium*, *Clostridium hiranonis,* and *Blautia*, all of which are associated with normobiosis (low dysbiosis index, Figure 3). The trend for the dysbiosis-associated bacteria (high dysbiosis index), however, was less clear, with a slight decrease in *Enterobacteriaceae* and an increase in *Streptococcus* after the race (Figure 3).

### 3.4. Metadata Associations

**Figure 4:**
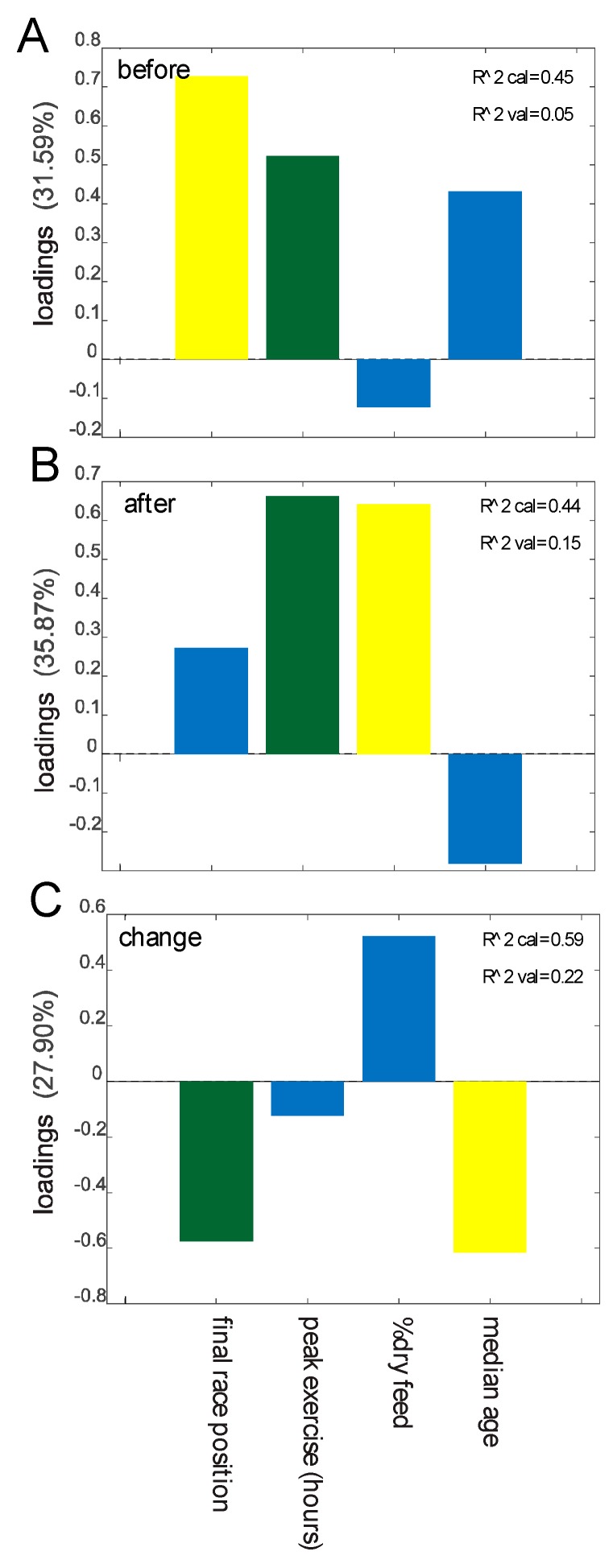
Prediction of dysbiosis based on metadata. Importance of predictors in the regression model for dysbiosis before the race (**A**), after the race (**B**), and changes from before to after the race (**C**). Predictors showing Spearman correlation *p* values <0.05 are colored green, *p* values <0.10 are yellow, and *p* values >0.10 are blue. Predictors with *p* values >0.10 in all cases are not included in the model.

We investigated possible associations between the data obtained from the questionnaires and stool consistency/parasite load (Tables S1 and S2) with the results from the analysis of the microbiota. Pre-race dysbiosis showed a positive association with poorer performance (lower finishing rank) and peak exercise (Figure 4A), while duration of peak exercise and percent dry feed (proportion of dry pellets in feed, by weight) showed the strongest positive association with post-race dysbiosis (Figure 4B). Changes in dysbiosis (from before to after the race) showed the overall strongest negative associations with poor performance and increasing age (Figure 4C).

No associations were found between either bacterial richness or alpha diversity measures with information obtained in the questionnaires, nor did we identify associations connected with fecal score or Giardia load two weeks before and after the race or dry feed brand (results not shown).

## 4. Discussion

**Figure 5:**
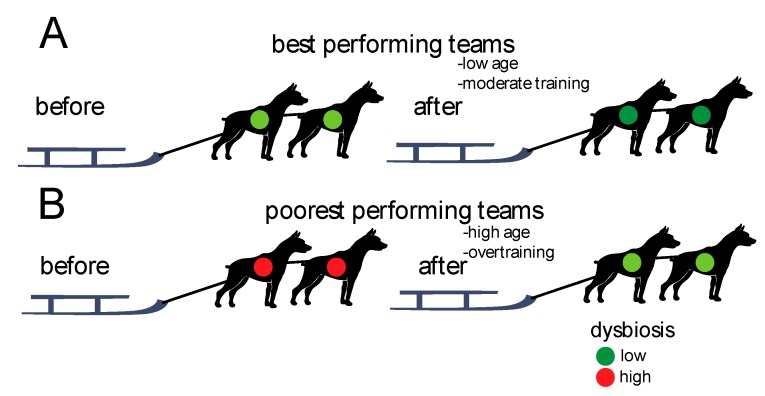
Schematic association between performances and dysbiosis. (**A**) The best performing teams showed the lowest degree of dysbiosis initially (before the race) and the lowest changes after the race. (**B**) The poorest performing teams showed the highest degree of dysbiosis initially (before the race) and the largest changes after the race.

We found that the teams with lowest levels of dysbiosis before the race showed the best performances. Furthermore, there was an overall decrease in dysbiosis from before to after the race, with the teams performing poorest showing the largest changes. The associations between gut microbiota dysbiosis and performances are summarized in Figure 5.

The main compositional structure was a negative association between *C. hiranonis* and *Enterobacteriaceae,* both before and after the race. As *C. hiranonis* is connected with normobiosis and *Enterobacteriaceae* with dysbiosis [6], the interactions between these bacteria could be important for the conditions in the gut.

Endurance racing is associated with damage to gut tissue due to oxygen depletion, with transient increases in gut leakage [7] and oxygen tension [17]. These conditions would normally favor growth of dysbiosis-associated bacteria, such as *Enterobacteriaceae* and lactic acid bacteria [11,18]. For the Femund Race, however, we observed a relative increase in the lactic acid bacterium *Streptococcus* after the race, while *Enterobacteriaceae* showed a slight decrease. A potential explanation for the lack of *Enterobacteriaceae* outgrowth could be that this genus of bacteria is negatively associated with *C. hiranonis*, which also showed a large increase after the race. Mechanistically, a negative association could be explained by *C. hiranonis* 7α-dehydroxylating activity [19]. This activity converts the primary bile acid, chenodeoxycholic acid, into the antimicrobial compound, lithocholic acid [20]. Elevated production of lithocholic acid during endurance racing [21] could also explain the observed reduced species richness from before to after the race.

Increased damage and oxygen leakage in the guts of the dogs participating in teams performing less well may lead to a greater growth potential of dysbiosis-associated bacteria. In order to prevent dysbiosis, we hypothesize that the dysbiosis-associated bacteria must be suppressed by a concordant increase in normobiosis-associated bacteria. This provides a potential explanation for the apparently contradictory results, with the teams performing less well showing the greatest decrease in dysbiosis after the race.

Gut leakage may also occur under circumstances of overtraining and poor performance [22]. Under conditions of chronic gut leakage, the gut microbiota may reach a tipping point with a new state of equilibrium in which the dysbiosis-associated bacteria predominate [3]. This could result in a more chronic dysbiotic state of the gut microbiota [23], potentially explaining why the poorer performing teams showed a higher degree of dysbiosis than the best performing teams prior to the start of the race.

Older age and poor performance were confounded with respect to association with dysbiosis. Results from previous studies, based on plate counts, have shown that there is an increase in streptococci in older dogs (>11 years) [24], and, at very high ages (~17 years), there is an increase in *Enterobacteriaceae* [25], both of which are associated with dysbiosis. Interestingly, we observed an increase in *Streptococcus*, but not *Enterobacteriaceae,* after the Femund Race. This may indicate that the post-race conditions in the gut may resemble that of increased age. At very high ages, however, the increase in *Enterobacteriaceae* may indicate dysfunction in resilient mechanisms, thereby resembling dogs with inflammatory bowel disease with increased *Enterobacteriaceae* and depleted *C. hiranonis* [2].

Ideally, all teams participating in the race would have been included in the study, but with limited resources available, we were obliged to select only a few of the teams. Unfortunately, four teams withdrew from the study at a timepoint when it was not possible to include additional teams. The small sample size limits the interpretative value of our results, and further investigation on this topic is warranted. Although we recorded stool consistency prior to and after the race, we lack information during the race. However, sled dogs participating in long-distance racing are known to have a high prevalence of diarrhea [26]. It should also be noted that many other confounding factors, such as heart function, team equipment, and the skills of the mushers, are associated with race performance and could have affected our results. In particular, diet could be a contributing factor for performance and team-specific clustering of the microbiota. More detailed information on some aspects, such as the concentration of energy, nutrients, and antioxidants in the diet, should have been obtained in order to address the effects of feed properly. Feed certainly also interacts with the gut microbiota, so, without detailed knowledge about feeding regimes, we cannot rule out that our results reflect the effects of feed [27]. However, since we do not have detailed information about the types of feed, it is difficult to compare our study with other studies focusing on feed effects [28]. Therefore, the relatively few parameters investigated in our study is a clear weakness. More considerations should be included in future studies, including more animals, recording the occurrence of diarrhea during the race, and more details about the feeding regimes.

Finally, our study was based on rectal swabs; ideally, more representative samples should have been obtained to reflect the gut microbiota. It is also possible that the storage of the samples directly after collection (freezing at −20 °C) before transport in a cold box and then freezing at -80℃ was not optimal for preservation of DNA, but this was the same for all samples collected at the race. Although rectal swabs have been shown to give reliable results in studies of the intestinal microbiota in humans [29], direct comparison of the results from our study with those of other studies may be difficult due to the major influence of extraction method on composition and diversity [30,31].

## 5. Conclusions

This study provides insights on the effects of extreme endurance exercise on the intestinal microbiota of dogs. We propose that normobiosis-associated bacteria could be a part of resilience mechanisms that are important for maintaining normobiosis during endurance racing.

## Supplementary Materials

The following are available online at https://www.mdpi.com/2076-2615/10/2/204/s1: Table S1: Association between dysbiosis and data from questionnaires, Table S2: Fecal Giardia load and fecal score from each attending dog two weeks before and two weeks after the race.

## Abbreviations

ANOVA	analysis of variance
OTU	operational taxonomic unit
PCR	polymerase chain reaction
PC	principal component,
PCoA	principal components analysis
std:	standard deviation
