# Supplementary material for: Pre- and Post-Race Intestinal Microbiota in Long-Distance Sled Dogs and Associations with Performance
Source: Animals (Basel). 2020 Jan 25;10(2):204. doi: 10.3390/ani10020204 (PMC7071093; doi:10.3390/ani10020204)
Supplement: Supplementary file 1 [file animals-10-00204-s001.pdf]

SUPPLEMENTARY INFORMATION

Suppl. Table 1. Association between dysbiosis and data from questionnaire

Kennel#	No. of dogs in the race	Median Dysbiosis	Final race pos	No. of dogs in kennel	No. of dogs< 1 year[1]	No. of dogs 1-3 year[2]	No. of dogs > 3 year[3]	No. of new dogs last year[4]	Peak exercise (hours)[5]	Dry feed (%)	Dry feed type	Median age (years)
1	12	-8.33	34	23	0	8	15	0	25	25	Sportsman's pride	5.50
2	11	-8.95	46	21	1	8	12	1	30	25	Royal Canine 5400	4.50
3	12	-5.86	58	12	0	5	7	1	40	50	Troll	6.50
4	11	-6.67	RD	29	0	18	11	1	80	50	Troll, extreme	3.50
5	11	-7.56	42	19	0	8	11	2	36	25	Labb	5.00
6	12	-9.22	24	15	5	6	4	0	24	25	Eukanuba performance	3.75
7	8	-4.67	63	11	0	3	8	2	60	25	Eukanuba performance	6.00
9	12	-8.03	24	10	0	2	8	0	30	50	Various	6.00
10	12	-7.07	RD	15	3	3	9	0	35	50	Troll	3.50
11	12	-6.89	8	12	0	9	3	6	48	25	Eukanuba working endurance	2.00
13	10	-8.13	22	12	0	7	5	8	36	25	Labb extreme	2.00
16	11	-5.89	65	9	0	3	6	3	35	50	Troll	2.00
18	10	-9.22	3	15	5	2	8	6	48	50	Appetitt extreme	3.00

20	12	-6.05	30	22	6	4	12	0	50	75	Sportsman's pride	4.00

[1-3] Number of dogs in in kennel based on defined age groups, [4] Number of new dogs introduced in kennel last year (2015) and [5] Number of hours at peak exercise before race. Teams that withdrew during the race are marked with RD.

Suppl. Table 2. Fecal *Giardia* load and fecal score from each attending dog two weeks before and two weeks after the race.

Kennel #	Dog ID	Age (yr)	Gender	Breed	*Giardia* IMS/IFAT[1]		Ave fecal score[2]	
					Pre-race	Post-race	Pre-race	Post-race
1	FL1.1	7	M	AH	0	1	4	4
	FL1.2	9	M	AH	0	1	3	3
	FL1.3	4	M	AH	1	0	2	3
	FL1.4	5	M	AH	0	0	4	4
	FL1.5	2	M	AH	0	1	3	3
	FL1.6	7	M	AH	0	0	2	3
2	FL2.1	4	M	SH	1	1	2	3
	FL2.2	7	M	SH	0	0	3	3
	FL2.4	4	M	SH	1	0	3	4
	FL2.5	3	F	SH	0	0	2	2
	FL2.6	6	M	SH	1	0	4	2
3	FL3.1	10	M	AH	1	0	4	3
	FL3.2	10	M	AH	0	0	2	2
	FL3.3	5	F	AH	0	0	2	2
	FL3.4	5	F	AH	1	0	3	3
	FL3.5	5	F	AH	1	1	3	2
	FL3.6	8	F	AH	0	0	3	3

4	FL4.1	3	M	AH	0	1	3	3
	FL4.2	3	F	AH	0	1	3	3
	FL4.3	3	M	AH	0	0	3	3
	FL4.4	8	F	AH	1	0	2	3
	FL4.5	6	M	AH	0	1	2	3
	FL4.6	4	M	AH	0	0	3	3
5	FL5.1	8	M	AH	0	0	3	2
	FL5.2	6	M	AH	1	0	2	2
	FL5.3	4	M	AH	0	1	3	3
	FL5.4	7	M	AH	0	1	3	3
	FL5.5	3	F	AH	1	0	3	3
	FL5.6	4	F	AH	0	1	2	3
6	FL6.1	3	F	AH	1	1	4	3
	FL6.2	3	M	AH	0	1	4	2
	FL6.3	5	F	AH	0	1	3	4
	FL6.4	7	M	AH	1	0	4	3
	FL6.5	7	M	AH	0	1	3	3
	FL6.6	3	F	AH	0	0	4	3
7	FL7.1	7	M	AH	0	0	3	3
	FL7.2	7	F	AH	0	0	2	3
	FL7.3	5	M	AH	1	0	4	2
	FL7.4	7	M	AH	1	0	3	2
	FL7.5	2	F	AH	1	0	2	2
	FL7.6	2	F	AH	0	1	4	3
9	FL9.1	5	F	AH	1	1	3	3
	FL9.2	5	F	AH	1	1	3	4

	FL9.3	6	F	AH	0	1	3	4
	FL9.4	6	F	AH	0	0	3	3
	FL9.5	6	M	AH	1	0	3	3
10	FL10.1	3	M	SH	1	1	2	3
	FL10.2	4	M	SH	0	1	3	4
	FL10.3	5	M	SH	1	1	3	3
	FL10.4	7	M	SH	1	1	3	3
	FL10.5	3	F	SH	1	1	3	4
	FL10.6	3	F	SH	1	ND	3	4
11	FL11.1	2	M	AH	1	1	4	4
	FL11.2	5	M	AH	1	1	3	4
	FL11.3	2	M	AH	0	1	4	4
	FL11.4	2	M	AH	1	0	4	4
	FL11.5	2	M	AH	1	0	4	4
	FL11.6	2	M	AH	0	0	3	4
13	FL13.1	2	M	AH	0	ND	4	ND
	FL13.2	7	M	AH	0	0	4	3
	FL13.3	4	M	AH	0	0	4	4
	FL13.4	2	M	AH	0	ND	4	ND
	FL13.6	2	M	AH	0	0	4	2
16	FL16.1	5	F	AH	1	1	4	3
	FL16.3	2	F	AH	0	1	3	3
	FL16.4	6	M	AH	0	1	3	4
	FL16.5	2	M	AH	1	ND	3	ND
	FL16.6	4	M	AH	0	1	3	2
18	FL18.1	4	M	AH	0	1	3	3

	FL18.2	2	M	AH	1	1	3	3
	FL18.3	3	M	AH	0	1	2	2
	FL18.4	3	M	AH	0	0	3	3
	FL18.5	3	M	AH	0	0	3	3
20	FL20.1	5	M	AH	0	0	2	3
	FL20.2	6	M	AH	0	0	2	4
	FL20.3	4	F	AH	0	0	3	3
	FL20.4	4	F	AH	0	1	2	3
	FL20.5	4	F	AH	0	0	3	2
	FL20.6	4	F	AH	0	0	3	3

[1]0=*Giardia*-negative, 1=*Giardia*-positive, ND=Not determined, M=male, F=female, AH=Alaskan Husky, SH=Siberian Husky

[2]Average of three measurements
